# Applicability of somatic monitoring instructions in clinical practice guidelines on antipsychotic drug use

**DOI:** 10.1186/s12888-021-03162-w

**Published:** 2021-04-12

**Authors:** Jurriaan M. J. L. Brouwer, Erien Olde Hengel, Arne J. Risselada, Eric N. van Roon, Hans Mulder

**Affiliations:** 1 Department of Clinical Pharmacy, Wilhelmina Hospital Assen, Mailbox: 30.001, Assen, Drenthe 9400 RA The Netherlands; 2 GGZ Drenthe Mental Health Services Drenthe, Assen, Drenthe The Netherlands; 3 Department of Psychiatry, Research School of Behavioural and Cognitive Neurosciences, University of Groningen, University Medical Centre Groningen, Groningen, The Netherlands; 4 Department of Pharmacotherapy, -Epidemiology & -Economics, Pharmacy and Pharmaceutical Sciences, University of Groningen, Groningen, Groningen The Netherlands; 5 Department of Clinical Pharmacy and Clinical Pharmacology, Medical Centre Leeuwarden, Leeuwarden, Friesland The Netherlands; 6 Department of Psychiatry, Interdisciplinary Centre for Psychopathology and Emotion Regulation, University of Groningen, University Medical Centre Groningen, Groningen, Groningen The Netherlands

**Keywords:** SIM, Applicability, Somatic, Monitoring, Antipsychotic

## Abstract

**Background:**

Clinical practice guidelines (CPGs) recommend the monitoring of somatic parameters in patients treated with antipsychotic drugs in order to detect adverse effects. The objective of this study was to assess, in adult and (frail) elderly populations, the consistency and applicability of the somatic monitoring instructions recommended by established CPGs prior to and during antipsychotic drug use.

**Methods:**

A search for national and international CPGs was performed by querying the electronic database PubMed and Google. Somatic monitoring instructions were assessed for adult and (frail) elderly populations separately. The applicability of somatic monitoring instructions was assessed using the Systematic Information for Monitoring (SIM) score. Somatic monitoring instructions were considered applicable when a minimum SIM score of 3 was reached.

**Results:**

In total, 16 CPGs were included, with a total of 231 somatic monitoring instructions (mean: 14; range: 0–47). Of the somatic monitoring instructions, 87% were considered applicable, although critical values and how to respond to aberrant values were only present in 28 and 52% of the available instructions respectively. Only 1 CPG presented an instruction specifically for (frail) elderly populations.

**Conclusions:**

We emphasize the need for a guideline with somatic monitoring instructions based on the SIM definition for both adult and (frail) elderly populations using antipsychotic drugs. In addition, CPGs should state that clear agreements should be made regarding who is responsible for interventions and somatic monitoring prior to and during antipsychotic drug use.

**Supplementary Information:**

The online version contains supplementary material available at 10.1186/s12888-021-03162-w.

## Background 

In both adult and (frail) elderly populations, antipsychotic drugs are used effectively in the treatment of several psychiatric disorders, such as schizophrenia, bipolar disorder, psychotic depression, and resistant major depression. In the (frail) elderly, antipsychotic drugs are also used, for example, in the treatment of delusional disorders and behavioural symptoms, despite limited evidence of effectiveness [1–5]. Treatment with antipsychotic drugs is associated with major adverse effects, such as metabolic disturbances including glucose intolerance, weight gain, cerebrovascular events, and extrapyramidal symptoms. These adverse effects contribute to a shorter life expectancy in both adult and (frail) elderly populations. In order to detect and treat antipsychotic-induced adverse effects, structural and frequent somatic monitoring is needed [6].

Several clinical practice guidelines (CPGs) recommend monitoring somatic parameters during treatment with antipsychotic drugs in order to detect antipsychotic-induced adverse effects. Furthermore, to be applicable in daily clinical practice, CPGs have to provide information regarding which monitoring parameters must be determined and when this should occur. It should be clear how often monitoring is necessary and what reference values are used together with recommendations for aberrant outcome values. It should also be clear whether these recommendations are applicable for specific patient populations like the (frail) elderly.

Several studies have shown that adherence to monitoring guidelines for antipsychotic drugs is poor. This may lead to underdetection of drug-induced adverse effects [7–10]. Adherence may be improved when monitoring instructions are clear and applicable, because clear guidelines are necessary to encourage healthcare professionals to implement proper monitoring practices. Several instruments are available to quantify whether CPGs are applicable and clear. The AGREE II instrument is a tool to assess the general quality of CPGs. Several studies using the AGREE II instrument have shown that many guidelines for the treatment of psychiatric disorders are not applicable and clear [11–13]. Although domains 4 and 5 of the AGREE II instrument assess the “clarity of presentation” and “applicability” of the CPG respectively, the items within these domains are not entirely suitable for assessing the quality of somatic monitoring instructions in CPGs specifically. The resulting scores per item do not sufficiently indicate what is causing the recommendations to be unclear or inapplicable.

For this purpose, the Systematic Information for Monitoring (SIM) score tool has been developed, in which every monitoring instruction is assessed on six different items. Several studies have used the SIM score to assess the quality of somatic monitoring instructions in CPGs. For example, Nederlof et al. assessed the clarity and applicability of somatic monitoring instructions, as stated in established CPGs for treatment of bipolar disorders, for patients using lithium. This led to the conclusion that an improvement in applicability is needed [14–16].

To our knowledge, it is unknown whether somatic monitoring instructions for the use of antipsychotic drugs are recommended consistently among different CPGs and whether they are applicable in daily clinical practice. Furthermore, it is unknown if CPGs provide specific information on somatic monitoring instructions for the (frail) elderly using antipsychotic drugs.

### Aim of the study

The objective of this study was to assess the consistency in CPGs of somatic monitoring instructions for antipsychotic-induced adverse events and whether these instructions are applicable in daily clinical practice for adult and (frail) elderly populations.

## Methods

### Identification and selection of clinical practice guidelines (CPGs)

We included both national and international CPGs for somatic monitoring instructions for adults (18–65 years old) and (frail) elderly patients using antipsychotic drugs. We defined frailty as “a condition in which the individual is in a vulnerable state at increased risk of adverse health outcomes and/or dying when exposed to a stressor” [17].

A search for national and international CPGs was performed by exploring the electronic database PubMed and a general search engine (Google).

Search terms were “antipsychotics”, “schizophrenia”, “bipolar disorders”, “dementia”, and “guidelines”. Criteria for the selection of CPGs for assessment were that the CPG 1) was clearly defined as a guideline, 2) was written in Dutch or English, and 3) included a section on antipsychotic treatment.

**Fig. 1:**
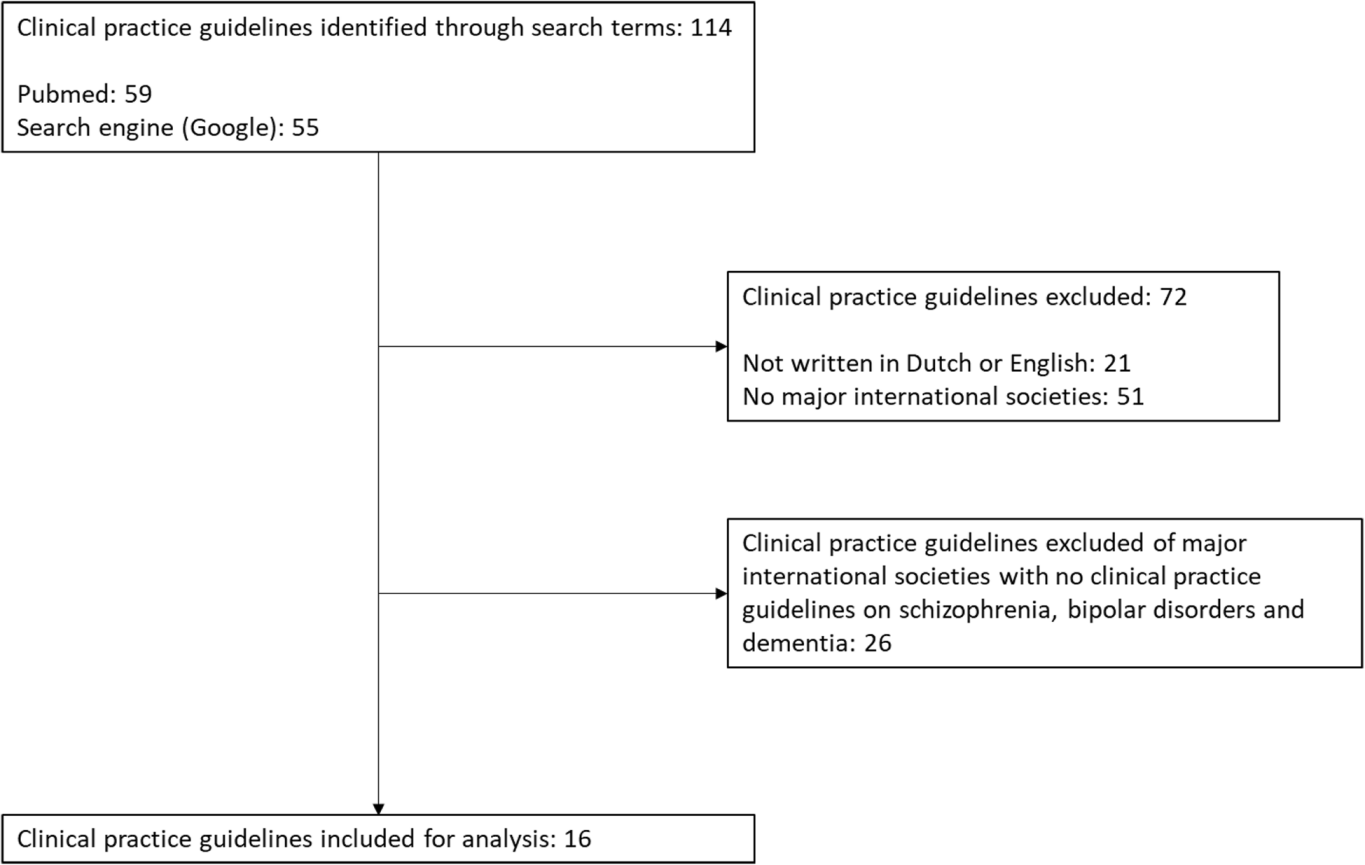
Selection of clinical practice guidelines (CPGs) flowchart

The search resulted in 114 CPGs of which 21 were not written in Dutch or English. CPGs were selected that were developed by major international societies with published CPGs on schizophrenia, bipolar disorders and dementia. The preselection procedure was conducted by consensus by HM and JB (Fig. 1).

### Assessment of somatic monitoring instructions

In order to assess the consistency of somatic monitoring instructions in different CPGs, each selected CPG was examined by authors EOH and JB for somatic monitoring instructions related to antipsychotic drug use. All available somatic monitoring instructions were collected from the CPGs. Somatic monitoring instructions of additional CPGs were collected whenever a referral was made within a selected CPG.

Somatic monitoring instructions were defined as instructions to measure a parameter prior to and during the use of antipsychotic drugs.

We defined a monitoring parameter as a parameter that can be measured as an anthropometrical parameter (e.g. height, weight, blood pressure, or waist circumference), a laboratory parameter (e.g. blood glucose, HbA_1C_, cholesterol, or prolactin) or a clinical monitoring parameter for indicating adverse effects (e.g. movement disorders).

Somatic monitoring instructions were classified as either a baseline monitoring instruction prior to the start of an antipsychotic drug or a monitoring instruction during maintenance therapy. When monitoring timing was not specified, the monitoring instruction was classified as both a baseline and a maintenance therapy monitoring instruction.

Somatic monitoring instructions were allocated either to mandatory or to recommended instructions based on how the monitoring instruction was formulated in the CPG.

Somatic monitoring instructions in CPGs were allocated to (frail) elderly psychiatric populations when these instructions were mentioned either in guideline sections specifically for (frail) elderly populations or in more general terms that every patient with the particular severe mental illness had to be monitored regardless of age.

### Applicability of somatic monitoring instructions

The applicability of somatic monitoring instructions was assessed using the SIM score [14–16]. The SIM score tool rates somatic monitoring instructions on the following six items: 1) what to monitor, 2) when to start monitoring, 3) when to stop monitoring, 4) how frequently to monitor, 5) critical values of the parameter, and 6) how to respond. Each item yields either a score of 0 (monitoring instruction is not clearly described) or a score of 1 (monitoring instruction is clearly described), resulting in a total SIM score varying from 0 to 6. Somatic monitoring instructions were considered applicable if they yielded an acknowledged minimal total score of 3 [16].

The authors believe that an important explanation for poor adherence to monitoring guidelines is the lack of information regarding which healthcare professional is responsible for the interpretation and treatment of aberrant monitoring outcomes. In order to address this issue, we focused on an additional item apart from the SIM score: who is responsible for treatment of aberrant monitoring outcomes. We did not add this item to the total SIM score because doing so would reduce the comparability of our results with those of other studies comparing CPGs with the SIM score.

The classification and assignment of the SIM scores of all somatic monitoring instructions was carried out by two authors independently (EOH and JB). Discrepancies were discussed with a third author (AR) until consensus was reached.

### Outcome measures

Outcome measures are the following: 1) an overview of recommended somatic monitoring instructions related to antipsychotic drug use per selected CPG; 2) the percentage of clearly described somatic monitoring instructions per selected CPG, outlined to each of the six SIM items and the additional item; and 3) the percentage of applicable somatic monitoring instructions per selected CPG.

### Data analysis

For each CPG, the number of somatic monitoring instructions were presented along with the SIM score items, corresponding mean/median SIM scores, and the percentage of somatic monitoring instructions that were applicable (SIM score ≥ 3).

## Results

The search resulted in 16 CPGs (see Supplementary Table 1, Additional file 1): 3 of either the American Psychiatric Association (APA) [18–20], the National Institute for Health and Care Excellence (NICE) [21–23], the Royal Australian and New Zealand College of Psychiatrists (RANZCP) [24–27], or the World Federation of Societies of Biological Psychiatry (WFSBP) [28–33] and 4 Dutch CPGs [34–37]. Of these 16 CPGs, three came from organizations based in North America, three from Oceania, three from worldwide organizations and seven CPGs were drawn up by organizations based in Europe.

Supplementary Table 2, Additional file 2 presents an overview of the somatic monitoring instructions in the included guidelines. All guidelines present within their scope that they were established either specifically for the adult population or for all patients with the particular disease. Additionally, the APA guideline for the treatment of schizophrenia [20] and the NICE guideline for the treatment of bipolar disorders [21] include a passage noting specifically that (frail) elderly patients “should be offered the same range of treatments and services as younger people”. There were no specific somatic monitoring instructions present for (frail) elderly populations, except for one cited in the APA guideline for the treatment of dementia [18] and related to blood count monitoring during the use of clozapine.

The SIM scores of the included guidelines are presented in Supplementary Table 3, Additional file 3. A total of 231 somatic monitoring instructions for people using antipsychotic drugs were found, with a mean of 14 somatic monitoring instructions per CPG. The number of somatic monitoring instructions per CPG ranges from no somatic monitoring instructions to 47 somatic monitoring instructions. This number includes somatic monitoring instructions from additional CPGs referred to in the included CPGs, corresponding with 23% (*n* = 52) of the total number of somatic monitoring instructions.

In 91% of the present somatic monitoring instructions, “what to monitor” is specified. “When to start monitoring”, “when to stop monitoring”, “how frequently to monitor”, “critical value”, and “how to respond” are specified in 97, 84, 70, 28, and 52% respectively. Responsibility for monitoring and/or for treatment of aberrant outcome values is specified in 56% of the present somatic monitoring instructions. A SIM score of at least 3 was reached in 87% of the present somatic monitoring instructions.

## Discussion

This study presents an overview of somatic monitoring instructions and their applicability in 16 CPGs regarding somatic monitoring prior to and during antipsychotic drug use by adult and (frail) elderly populations. Overall, most of the somatic monitoring instructions are applicable with a mean SIM score equal to or higher than 3. The included CPGs address several important aspects and achieve high scores on “what to monitor” (91%), “when to start monitoring” (97%), “when to stop monitoring” (84%), and “how frequently to monitor” (70%). However, the overall applicability of CPGs can be further improved in the following ways. First, by including information about critical values (currently 28%), how to respond to aberrant outcome values in terms of interventions (currently 52%), or which healthcare professional is responsible for these interventions (currently 56%).

Second, there is no consensus on what parameters must be monitored prior to and during the use of antipsychotics. Four CPGs provide instructions for around 30 or more monitoring parameters, while another 8 provide no information. Four remaining CPGs are situated in between, providing around 15 to 27 monitoring parameters.

Finally, with one exception, none of the guidelines provide specific somatic monitoring instructions regarding the monitoring of (frail) elderly populations using antipsychotics drugs.

### Applicability

Although somatic monitoring instructions indicate which parameter should be monitored and how often, they lack information regarding the presentation of critical values, how to respond to aberrant outcome values, and whose responsibility it is to monitor and to intervene based on aberrant outcome values. The lack of information regarding whose responsibility it is to initiate monitoring may contribute to the low monitoring rates among psychiatric populations [38], as psychiatrists and primary health care professionals express different opinions regarding who is responsible for the monitoring and treatment of, for example, metabolic effects due to psychotropic drugs [39–41]. In addition, the lack of clarity in how to respond in terms of interventions and who is responsible to intervene may be a contributing factor in the inadequate treatment of psychiatric patients in the case of, among other things, metabolic abnormalities [38, 42].

For example, Bruins et al. present persistently low treatment rates for metabolic abnormalities among patients with psychotic disorders, despite reasonable annual monitoring rates during a period of 3 years. More than half of the patients for whom pharmacotherapy for metabolic disorders was recommended did not receive any treatment [42]. Although the authors did not investigate the reason for these alarmingly low treatment rates, a contributing factor could be the lack of information in available guidelines for interventions in the case of aberrant outcome values.

The study by Bruins et al. was performed in the Netherlands. The Dutch guidelines contain neither information on interventions in the case of aberrant outcome values nor information on responsibility for treating metabolic abnormalities. Possibly, adding mandatory statements that manage outcomes of monitoring as part of integrative care should be addressed.

### Consensus

The number of instructions and the recommended frequency of monitoring differs significantly between CPGs. Although somatic monitoring among patients with severe mental illness is recognized internationally, some CPGs do not specifically recommend somatic monitoring during the use of antipsychotic drugs. For example, the APA guideline for the treatment of bipolar disorders and the NICE guideline for the treatment of dementia provide no instructions regarding somatic monitoring at all. In addition, CPGs that do present somatic monitoring instructions differ in the amount of instruction. This indicates a lack of consensus on what to monitor prior to and during antipsychotic drug use. This lack of consistency among guidelines may have two causes.

First, the reason for the lack of consistency among guidelines might be the scope of the guideline. Guidelines in which treatment with antipsychotic drugs is only (a minor) part of the treatment protocol probably provide less information on monitoring. This suggestion seems correct in view of the lower rate of somatic monitoring instructions in guidelines for dementia.

A second reason for the lack of consistency among guidelines might be the scarcity of evidence for the positive effects of structural monitoring and interventions in the case of aberrant outcome values. Recommendations in CPGs are preferably evidence-based. For example, (frail) elderly are often excluded from research resulting in hardly any evidence for monitoring in these patients. The lack of evidence results in recommendations for this population that are mostly empirical or based on consensus and therefore guideline committees will make different considerations for which somatic monitoring instructions should be part of a guideline [43–45].

The results imply that there is no uniformity among CPGs regarding the content and number of somatic monitoring instructions prior to and during the use of antipsychotic drugs among adult and (frail) elderly populations. To standardize somatic monitoring instructions in CPGs, there is a need for consensus statements. In addition, more research is necessary to investigate the associations between the monitoring, prevention, and treatment of antipsychotic-induced adverse effects. In lack of evidence, consensus statements can be established by using, for example, the Delphi method. This method is used to reach consensus by sending questionnaires about a specified topic to a panel of experts. The response to these questionnaires is aggregated and send to the other experts. Experts can adjust their answers in another round based upon the answers of the group. The intended outcome after several rounds is a consensus about the topic supported by all experts. A good example of a consensus statement established with the Delphi method was published recently regarding the monitoring of lithium [46, 47].

### Somatic monitoring instructions for (frail) elderly populations

This study shows that CPGs, including those for patients with dementia, hardly recommend specific somatic monitoring instructions for (frail) elderly populations using antipsychotic drugs. This might be explained by the lack of research regarding antipsychotic-induced adverse effects in (frail) elderly populations.

However, a systematic review and meta-analysis by Schneider-Thoma et al. indicated a higher risk of second generation antipsychotic-induced somatic serious adverse events in older populations compared to adult populations [48]. Because antipsychotic drug treatment in (frail) elderly populations should be safe, it seems reasonable to monitor according to the monitoring recommendations developed for adult patients. However, discussions may arise as to what to monitor and how frequently to monitor in elderly populations, especially when the frail and vulnerable elderly with short life expectancy are taken into account. For example, it can be argued that the measurement of blood glucose is still necessary in the (frail) elderly to prevent short-term complications, whereas the monitoring of blood lipids seems less necessary, given its association with longer-term complications in a population with a short life expectancy [49, 50].

The lack of specific somatic monitoring instructions for the (frail) elderly using antipsychotic drugs may affect medication safety. For example, Ndukwe et al. show that glucose monitoring was suboptimal in (frail) elderly patients using antipsychotic drugs, putting these patients at risk for hyperglycemia [9]. To address these issues, it is advisable to incorporate a specific section in current and future guidelines regarding monitoring instructions for relevant subpopulations, including the (frail) elderly, according to the SIM definitions.

A few limitations should be mentioned. Although the SIM score tool addresses the applicability of both the somatic monitoring instructions that are present and, therefore, the applicability of the clinical practice guideline, it does not assess the clinical relevance of the presented somatic monitoring instructions. Furthermore, in order to analyze a manageable amount of CPGs we used an arbitrary search strategy. We have based our results upon the selection of CPGs from major societies (APA, NICE and RANZCP) from three continents, one global society (WFSPBP) and CPGs made in The Netherlands. We do not know whether these results are generalizable to, for example, smaller societies and CPGs in other languages than English or Dutch. However, despite this limitation, we analyzed 16 CPGs and therefore we believe that our results provide valuable information to other international societies to critically reflect upon their CPGs.

## Conclusions

In conclusion, this study shows that improvements are possible in the applicability of monitoring instructions for patients using antipsychotic drugs. Reference values, interventions in the case of aberrant outcome values, and responsibilities for treating aberrant values should be stated more clearly with respect to both adult and (frail) elderly populations. The need for somatic monitoring is recognized internationally. However, the evidence for the positive effects of somatic monitoring is scarce. More research is necessary to investigate the association between somatic monitoring and treatment outcomes in order to achieve more consensus on somatic monitoring instructions in CPGs.

## Supplementary Information

**Additional file 1: Supplementary Table 1.** Clinical Practice Guidelines.

**Additional file 2: Supplementary Table 2.** Clinical Practice Guidelines.

**Additional file 3: Supplementary Table 3.** Clinical Practice Guidelines.

## Data Availability

Datasets used and/or analyzed during the current study are available from the corresponding author on reasonable request.

## Abbreviations

AGREE: Appraisal of Guidelines for Research & Evaluation
APA: American Psychiatric Association
CPG: Clinical practice guideline
NICE: National Institute for Health and Care Excellence
RANZCP: Royal Australian and New Zealand College of Psychiatrists
SIM: Systematic Information for Monitoring
WFSBP: World Federation of Societies of Biological Psychiatry
